# A predictive signature for therapy assignment and risk assessment in prostate cancer

**DOI:** 10.18632/oncoscience.271

**Published:** 2015-11-20

**Authors:** Désirée Bonci, Ruggero De Maria

**Affiliations:** ^1^ Department of Hematology, Oncology and Molecular Medicine, Istituto Superiore di Sanità, Rome, Italy; ^2^ Scientific Directorate, Regina Elena National Cancer Institute, Rome, Italy

**Keywords:** prostrate cancer, non-coding RNA, miRNA, genes

## Abstract

Prostate cancer remains the second leading cause of death in men. It is imperative to improve patient management in identifying bio-markers for personalized treatment. We demonstrated miR-15/miR-16 loss and miR-21 up-regulation and deregulation of their target genes, which represent a promising signature for ameliorating therapy assignment and risk assessment in prostate cancer.

## DISCUSSION

**Figure 1:**
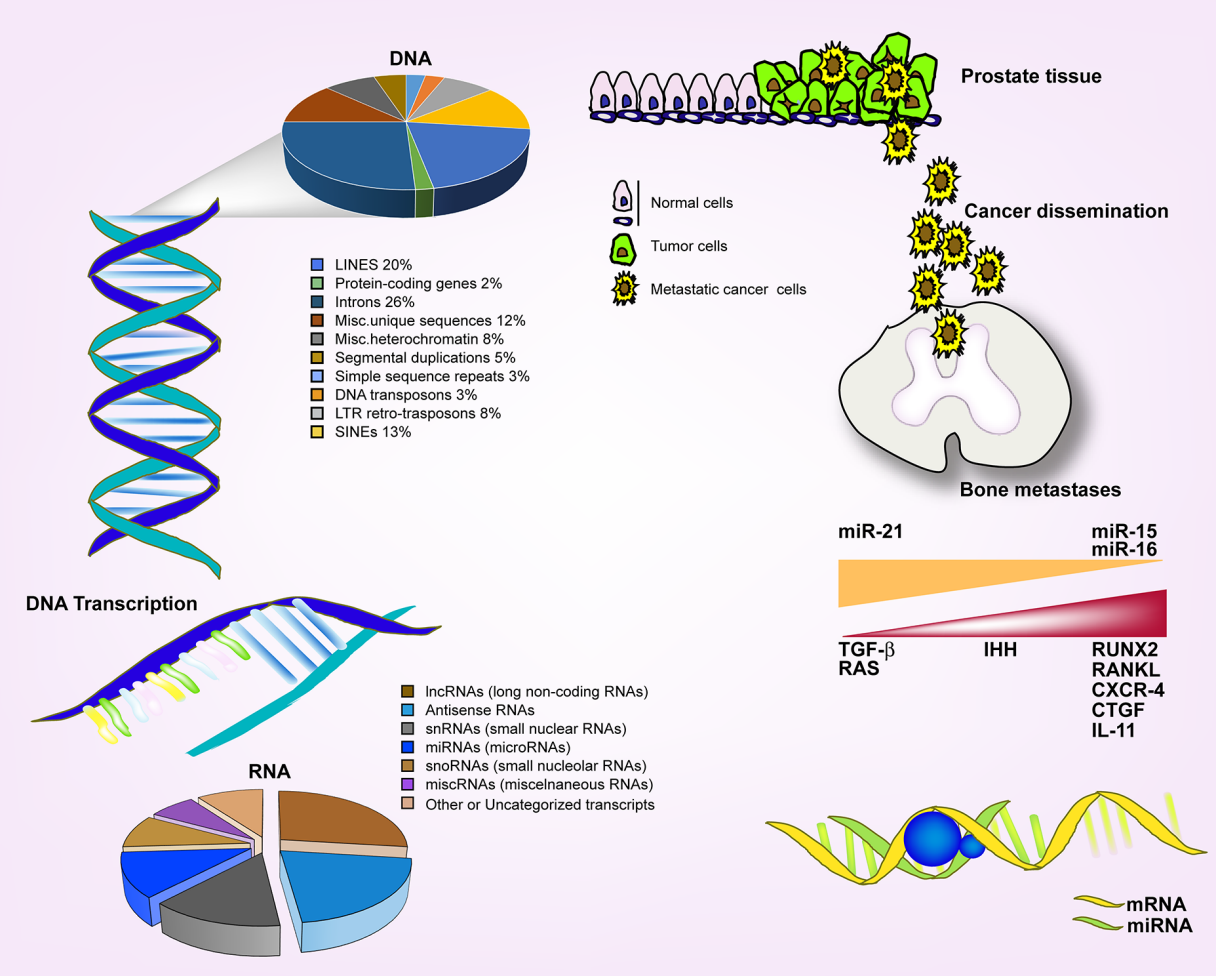
Pro-metastasis aberrant circuit involving TGF-β, RAS, IHH and miRNA alterations Genome representation following the international human sequencing consortium (2001). LINEs (long interspersed nuclear elements); SINEs (short interspersed nuclear elements). Cartoon representing the molecular mechanisms involved in bone metastasis dissemination. Transforming growth factor beta (TGF-β); Indian hedgehog (IHH); Runt-related transcription factor 2 (RUNX-2); Receptor activator of nuclear factor kappa-B ligand (RANKL); Interleukin 11 (IL-11); C-X-C chemokine receptor type 4 (CXCR-4); Connective Tissue Growth Factor (CTGF).

“Molecular Structure of Nucleic Acids: A Structure for Deoxyribose Nucleic Acid” was an article published by Francis Crick and James D. Watson in the scientific journal Nature in 1953. This article was termed a “pearl” of science because it contains the answer to a fundamental mystery about life, how genetic instructions are held inside organisms and how they pass from generation to generation. The discovery of the DNA double helix made clear that genes are functional parts of biological systems. As consequence, we have assumed the postulate that DNA contained genes which are transcribed in mRNA and then translated into proteins. Proteins could control genes in feedback loops then they were considered the major actors in the living system theatre. Francis Crick and James D. Watson at that time did not know that their extraordinary discovery was only showing one face of the moon due to limitations of technology. In fact, years after the advent of high-resolution whole genome and transcriptome sequencing technologies showed that there exists a second intriguing face. Results demonstrated the existence of coding and non-coding genes showing that at least 90% of the genome is actively transcribed in non-coding RNAs whereas protein coding genes (mRNA) represent < 2% of total sequences[1]. According to knowledge acquired until now, -who knows in the near future-, non-coding RNAs may be generally grouped into two major classes, small (18-200 nt) and long (200 nt to > 100Kb) RNAs based on transcript size[1]. Small RNAs include the well-documented microRNA (miRNA) gene family. Victor Ambros, Rosalind Lee and Rhonda Feinbaum discovered the first miRNA in 1993, lin-4, that was expressed in C. elegans[2]. In 2000, the small molecule lin-7 was discovered exhibiting silencing activity on lin-41[3]. One year later, the large family of microRNA was studied in C. elegans, Drosophila and “Homo Sapiens”[4]. This scientific revolution produced the maximum effect when a non-coding RNA perturbation was associated with disease development in humans including cancer[1,5]. An article published by Croce *et al.* in 2005 reported that miR-15 and miR-16 deletion caused CLL development and progression[6]. In 2008, the same miR-15/miR-16 cluster down-regulation was studied in solid tumors and was associated with prostate cancer progression[7]. Subsequently, several other articles demonstrated miR-15 and miR-16 are tumor-suppressor miRNAs[8]. Many articles focusing on the deregulation of miRNAs in cancer were published in the following years to come. The Oncomir miR-21 has been thoroughly investigated[9]. In prostate cancer, miR-21 promotes hormone-dependent and hormone-independent growth[10]. Over the last few years many studies have been devoted to elucidating the aberrant molecular mechanisms involving miRNAs and their multiple mRNA targets. Since non-coding RNAs are abundant and extremely stable in biological fluids they represent a new source for the discovery of reliable and sensitive biomarkers for optimizing diagnosis, prognosis and therapy-sensitiveness prediction of advanced patients. In fact, programs for patient management and risk assessment such as diagnostic screening, active surveillance protocols and clinical trials for new drug testing, are waiting for non-invasive tools and the best markers. In the era of personalized therapy, molecular markers are guiding the decision-making process regarding the best therapeutic treatment for approaching radical surgery and to select optimal sensitive-candidate-patients, which are the main goals. This is key in drawing the researchers' and the clinical community's attention. Up until today, we are obliged to consider and interpret a plethora of results and data produced in the last decade which have changed and renewed our point of view. DNA, non-coding RNA, mRNA and proteins act together in concert in a sophisticated equilibrium to maintain biological systems. In a pathological state, this equilibrium is destroyed and new aberrant networks are established. This is one of the reasons why the battle against cancer is still open. Thus, we have to approach cancer from a variety of sides in order to discover and block aberrant molecular mechanisms promoting cancer. Numerous genetic alterations have been associated with prostate cancer which remains one of the leading causes of death in men. Following the demonstration that miR-15 and miR-16 down-regulation was directly involved in prostate cancer progression, we embarked on discovering which multiple mechanisms could synergize and create the tumor phenotype[11]. We extended the study by demonstrating that an early K-RAS-modified tumor model assumed a metastatic phenotype after miR-15 and miR-16 down-regulation. Although not frequently mutated in prostate cancer, RAS isoforms play a pivotal role in multiple pathways that have been implicated in tumorigenesis. Thus, the results showed that miR-15/miR-16 down-regulation synergize with RAS activation in promoting tumor aggressiveness. On the other hand, RAS has been shown to promote prostate cancer progression by working synergistically with other pathways. In particular, a large body of literature indicated a collaboration between RAS and TGF-β, with a prominent role of RAS signaling in the conversion from anti-to pro-oncogenic TGF-β signaling. Hatley *et al*., showed that RAS aberrant cascade is reproduced by miR-21 over-expression. We revealed a simultaneous alteration of miR-15/miR-16 down-regulation and miR-21 up-regulation in a consistent fraction of patients' primary cells and tissues. Then, we studied the cross-talk of miR-15/miR-16 down-regulation and miR-21 up-regulation in cancer development. Increased miR-21 and loss of miR-15/miR-16 seem to particularly cooperate at the level of TGF-β signaling. Interestingly, miR-15 and miR-16 can target Activin RIIA, a receptor belonging to the TGF-β family triggered by Activin A and Nodal. The increased expression of Nodal reported in prostate cancer may therefore contribute to enhancing SMAD signaling after loss of miR-15 and miR-16. In addition, miR-21 controls SMAD-7, an inhibitor of TGF-β pathway. We showed a new molecular circuit driven by miR-15, miR-16 and miR-21 alterations, resulting in aberrant TGF-β signaling. In the bone marrow microenvironment, mesenchymal stem cells and metastatic prostate cancer cells can produce TGF-β, which is reported to play a significant role in prostate cancer progression, as indicated by its release in sera of advanced patients[12] and by the TGF-β inhibitors' ability in preventing the formation of bone metastasis in preclinical models[13]. Several bone metastasis-associated genes induced by TGF-β were revealed to be an indirect effect, such as RANKL, RUNX2, CXCR-4, CTGF and IL-11. It has been reported that TGF-β can post-transcriptionally regulate IHH ligand, a key gene in bone metastasis formation. We demonstrated that miR-15 and miR-16 can directly control IHH gene. Our results showed a pro-metastasis aberrant circuit involving TGF-β, RAS, IHH and miRNA alterations. There are many TGF-β signaling antagonist agents under development at both the pre-clinical and clinical stages. In patients with CRPC (castration-resistant prostate cancer) and bone metastases, Denosumab, targeting RANKL ligand, reduced the risk of skeletal complications. Increasing synthetic Hh antagonists are being reported in the literature. Several of these compounds are now in clinical trials, including GDC-0449. Our data demonstrate the function of miR-15, miR-16 and miR-21 together with the deregulation of direct and indirect gene targets in bone metastases (Figure 1) and may acquire importance as biomarkers for active surveillance protocols and in therapy decision-making for patient treatment.
